# Application of artificial intelligence as postoperative support for patients undergoing thoracic surgery

**DOI:** 10.1590/0100-6991e-2026000425-en

**Published:** 2026-04-09

**Authors:** BEATRIZ D’ÁVILA PEREIRA DA SILVA, ERNESTO EVANGELISTA, JOÃO ALÉSSIO JULIANO PERFEITO, ANDRÉ MIOTTO

**Affiliations:** 1 - Universidade Federal de São Paulo (UNIFESP), Escola Paulista de Medicina - São Paulo - SP - Brasil; 2 - Hospital São Paulo (Cirurgia Torácica) - São Paulo - SP - Brasil

**Keywords:** Surgery, Thoracic Surgery, Postoperative Care, Artificial Intelligence, Generative Artificial Intelligence, Cirurgia, Cirurgia Torácica, Cuidados Pós-Operatórios, Inteligência Artificial, Inteligência Artificial Generativa

## Abstract

**Introduction:**

Artificial intelligence (AI) is playing an increasingly significant role in medicine, from imaging-based diagnosis to personalized treatment. In the postoperative period, it may enhance patient support by answering questions more clearly and reducing the need for in-person visits. However, its reliability and effects on the patient experience remain insufficiently studied. This study examined the potential of AI as a complementary tool in the care of patients undergoing thoracic surgery, assessing response accuracy, patient acceptance, and its potential to reshape postoperative follow-up.

**Methods:**

Real patient questions were collected and answered by both ChatGPT and specialist physicians. The responses were compared in terms of clarity, accuracy, and completeness. Participants evaluated their satisfaction with the information provided, their need to seek additional details, and their comfort level with using AI without medical supervision.

**Results:**

Among the patients evaluated, 74.3% reported that ChatGPT fully addressed their questions, and 91.4% found the language clear and accessible. However, 62.8% still indicated a need for medical confirmation. In addition, 51.4% of participants stated that they would seek additional information even after receiving an AI-generated response.

**Conclusion:**

The findings suggest that AI has strong potential to enhance patients’ postoperative experience by providing rapid, accessible answers. However, its use should be integrated into a hybrid care model that combines technology with individualized medical oversight.

## RESUMO

**Introdução:**

A inteligência artificial (IA) tem desempenhado um papel cada vez mais significativo na medicina, desde o diagnóstico por imagens até tratamentos personalizados. No pós-operatório, essa ferramenta pode otimizar o suporte ao paciente, esclarecendo dúvidas e reduzindo a necessidade de consultas presenciais. No entanto, sua confiabilidade e impacto na jornada do paciente ainda são pouco explorados. Esse estudo explorou o potencial da IA como artifício complementar ao cuidado de pacientes submetidos a cirurgia torácica, analisando a precisão das respostas, aceitação pelos pacientes e potencial para transformar o acompanhamento médico.

**Métodos:**

Foram coletadas dúvidas reais de pacientes, que foram respondidas tanto pelo chatGPT quanto por médicos especialistas. As respostas foram comparadas quanto à clareza, acurácia e completude. Os participantes avaliaram a satisfação com as informações fornecidas, a necessidade de buscar mais detalhes e o conforto em utilizar a IA sem supervisão médica.

**Resultados:**

Dos pacientes avaliados, 74,3% relataram que suas dúvidas foram completamente solucionadas pelo chatGPT, enquanto 91,4% consideraram a linguagem clara e acessível. No entanto, 62,8% ainda expressaram a necessidade de confirmação médica. Além disso, 51,4% dos participantes afirmaram que buscariam informações adicionais mesmo após a resposta da IA.

**Conclusão:**

Os resultados apontam que a IA tem grande potencial para melhorar a experiência pós-operatória dos pacientes, oferecendo respostas rápidas e acessíveis. No entanto, sua aplicação deve ser integrada a um modelo híbrido de assistência, combinando tecnologia com individualização médica.

## INTRODUCTION

Artificial intelligence consists of the creation of computer programs capable of learning and reasoning like human beings to solve problems practically and creatively^1^. In medicine, this technology has already been incorporated into several aspects of daily professional practice^1^. Its ability to collect and analyze data is extremely useful for the management of medical records, the conduct of scientific research, and the identification of new medications^2^, allowing the integration of information from multiple centers and laboratories. 

At the end of 2022, the American technology company OpenAI launched ChatGPT^3^, a tool that gained global prominence by offering a new approach to interaction with artificial intelligence. Unlike traditional virtual assistants, ChatGPT is an advanced language model capable of understanding and generating text in more than 50 languages. Its broad accessibility, through a free interface available via website and application, combined with its ability to maintain interaction context and dynamically adapt to the information provided throughout the conversation, has contributed significantly to its widespread adoption^4^. Therefore, ChatGPT allows users to develop continuous and in-depth dialogues on the same topic^5^, making the search for information more direct and integrated, in contrast to conventional internet search methods, which are often fragmented and superficial. 

For patients, ChatGPT offers a wide range of utilities in the healthcare context, from providing accurate information about diseases, therapies, and medications to assisting in understanding their conditions and treatment options. In addition, it can be used to monitor symptoms over time, send medication reminders, provide mental health support, educate about healthy habits, and offer emotional support^5^ ^-^ ^7^. With the ongoing development of the technology, there are several promising applications on the horizon, such as AI-assisted diagnosis, personalization of treatments based on individual data, continuous health monitoring, enhancement of telemedicine, interactive health applications, and even risk alert and prevention systems^5^ ^,^ ^6^. One of the topics frequently studied in relation to artificial intelligence has been the prediction of postoperative adverse effects and the real-time assessment of complication risk, which may contribute to improved patient outcomes and postoperative recovery^8^.

In light of this context of limited evidence and ongoing debate, this study aims to validate ChatGPT as an appropriate platform for patient use, with the potential to improve the postoperative hospitalization experience, based on the evaluation of the following criteria: truthfulness, content relevance, accessible language, need for medical supplementation, and comfort in using the tool without supervision by a health care professional.

## METHODS

All patients aged 18 years or older who were admitted to the Thoracic Surgery ward of Hospital São Paulo (HSP), underwent a surgical procedure, and accepted and agreed to the informed consent form (Termo de Consentimento Livre e Esclarecido - TCLE) in the year 2024 were included. Patients who did not meet any of these criteria were excluded. Participants were approached by the researcher and invited to take part in the study by completing two forms: one to report their questions and another to evaluate the response provided by the AI. 

Form 1 was administered to collect patients’ main questions, which were then entered into the artificial intelligence application. The responses obtained were reviewed and validated by health care professionals and subsequently presented to the patients. Using Form 2, patients evaluated the adequacy and clarity of the responses, the need to seek additional information, and their sense of security when using the artificial intelligence platform.

Comparisons between two qualitative variables were performed using the chi-square test. Spearman’s test was used to assess the correlation between age and satisfaction with the response. For all statistical tests, the significance level adopted was a type I error, with a p value <0.05.

The Institutional Research Ethics Committee approved this research protocol under CAAE: 76929724.4.0000.5505, Opinion No. 7.003.211. This study was conducted in full compliance with all Good Clinical Practice Policies and Procedures, as well as all applicable national laws and regulations, including Resolution No. 466/2012 of the National Health Council. Data collection, recording, and reporting were accurate and ensured the privacy, health, and well-being of research participants during and after the study.

## RESULTS

The sample size consisted of 85 (eighty-five) participants. Of these, 49 (forty-nine) patients agreed to participate in the study, and 35 (thirty-five) reported questions and evaluated the application’s responses using Form 2, as shown in Flowchart 1.

**Flowchart 1:**
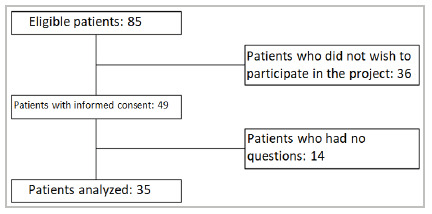
Sample flow

The AI intervention was conducted using the ChatGPT model (GPT-5), provided by OpenAI and accessed through its public free version (chat.openai.com) between March and April 2025. All interactions were conducted in Portuguese, in a web-based environment, using a personal tablet, ensuring anonymity. Standardized prompts were used, beginning with a description of the clinical context and the patient’s question (e.g., “I had thoracic surgery and I have a few questions”), maintaining temperature =0.7 and the default maximum response limit. The scope of the intervention was strictly educational, with no diagnoses, management recommendations, or clinical decision-making. 

The primary outcome was the accuracy of ChatGPT regarding the information conveyed to the patient and the patient’s understanding, as presented in Table 1 and detailed for each question below. Secondary outcomes were operationally defined as follows:

- “Question resolved” - when the participant selected a score of 4 or 5 on the Likert scale (1-5) for the item “Did the answer resolve your question?”
- “Clarity of language” - when the participant selected 4 or 5 on the item “Was the answer clear and easy to understand?”
- “Need for medical confirmation” - when the participant selected 1 or 2 on the item “Would you feel the need to confirm the answer with the doctor?”

Secondary outcomes were assessed immediately after reading the responses, using Form 2.

**Table 1:** Percentage results from Form 2

	YES	NO	PARTIAL
Did the question get resolved?	74.29%	2.86%	22.86%
Would you seek additional information?	51.43%	48.57%	-
Did it provide extra information?	51.43%	42.86%	5.71%
Was the language accessible?	91.43%	0.0%	8.57%
Would you ask for aphysician’s opinion?	62.86%	37.14%	-
Comfortable using it without medical supervision?	54.29%	20.00%	25.71%

### Satisfaction with ChatGPT responses

Among the patients evaluated, most reported that their questions were fully resolved after ChatGPT’s response. Others stated that the response was partially satisfactory, while a small proportion indicated that their questions were not resolved. 

### Need to seek additional information

Half of the patients indicated that they would still seek additional information after receiving the ChatGPT response, while the remaining participants stated that the answer was sufficient to clarify their questions. 

### Details of the information provided

More than half of the patients reported that the application provided additional details, and most indicated that they appreciated the extra information. Of the 15 individuals who stated that ChatGPT answered only what it was asked, only 1 would have liked to receive additional information, compared with 40% who agreed that the application provided an appropriate level of detail.

### Clarity of language

The vast majority of participants considered ChatGPT’s language easy to understand. However, some reported difficulty with certain terms used in the responses. No patient reported being unable to understand the application’s answer. 

### Medical confirmation

Most patients indicated that they would still like a physician’s opinion, whereas the others stated that the ChatGPT response was sufficient to address their questions. 

### Comfort in using AI without medical supervision

Among the patients evaluated, more than half reported that they would feel comfortable receiving ChatGPT responses without supervision by a health care professional. Others stated that they would not feel comfortable and would prefer a physician follow-up to verify the responses, while the remaining participants reported being partially comfortable resolving their questions independently.

Statistical analysis was performed using IBM SPSS Statistics, version 29. Proportions and their respective 95% confidence intervals (95% CI) were described. Comparisons between categorical variables used Pearson’s chi-square test, with a pre-established significance level of p <0.05. Denominators were standardized according to the number of patients who fully completed Form 2.

A comparative analysis was conducted among study variables to identify statistically relevant correlations (i.e., with a significance level of p<0.05). Among the analyses performed, it was not possible to establish a statistical correlation between “age” and “satisfaction with the response” (p=0.176). In addition, the study was also unable to relate the type of “surgical procedure” to “satisfaction with the response” (p=0.976).

**Figure 1:**
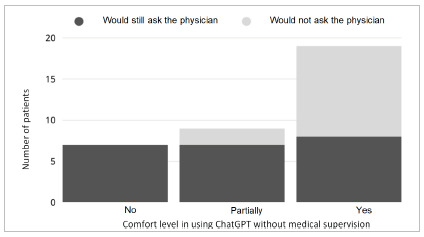
Relationship between “seeking the physician’s opinion” and “comfort using ChatGPT”

**Figure 2:**
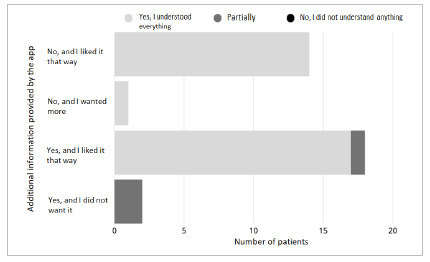
Relationship between “clarity of language” and “amount of information provided by ChatGPT”

In contrast, it was possible to establish a statistically significant correlation between the variables “seeking the physician’s opinion” and “comfort using ChatGPT” (p=0.017), indicating that patients who would still ask their physician tend to feel uncomfortable using the application without supervision by a health care professional, as shown in Figure 1. Another correlation was found between “amount of information provided” and “clarity of language” (p=0.00004), demonstrating that patients who considered the responses excessive or containing unnecessary information had greater difficulty understanding the language used, as presented in Figure 2.

Regarding ethical aspects and data protection, all interactions with the model were anonymous and did not involve the transmission of sensitive data. No personal information, identifiable clinical information, or medical record number was shared with the system. The generated responses were not stored on external servers, and records (logs) were kept locally in an encrypted spreadsheet, with access restricted to the research team. The study followed the ethical principles of the Declaration of Helsinki and the guidelines of the LGPD (Law No. 13,709/2018). 

## DISCUSSION

The findings of this study suggest that the primary factor for understanding and acceptance of artificial intelligence as a supportive tool is the patient’s openness to receiving information through this platform. Patients who appreciate additional details and are interested in further researching what was provided are more likely to feel comfortable with the technology, allowing AI to function as a third participant in the physician-patient relationship^9^, contributing to care and helping to overcome the language barrier created by medical jargon^5^. Health care professionals are key to ensuring the relevance of AI in medical care, as well as its application in everyday practice^10^.

Although most participants had their questions resolved, many still consider medical input indispensable. It was observed that the AI platform would be highly useful as a complementary and additional tool, allowing patients to access it at any time to obtain information, better understand what to ask their physician during the consultation, and follow the recovery process, as well as the next steps of the therapeutic plan. In addition, the limitations and ethical dilemmas inherent to this technology must be taken into account^5^ ^,^ ^11^. Chatbots such as ChatGPT have difficulty verifying the reliability of information sources, which can make them tools that replicate inaccuracies and undermine their use as information systems. Other limitations include copyright infringement, legal complications, and imprecise concepts^5^. 

Therefore, the study supports the current literature on the use of artificial intelligence in health care. Although there are still a few studies documenting the different aspects of ChatGPT in the health care field^5^, prior research has shown that AI-based tools can be effective in providing medical information, but public acceptance may vary depending on perceived reliability and clarity of the responses^11^. At times, patients use scientific terms that they know only because of their experience with their clinical condition, which leads ChatGPT to respond with a similar level of medical jargon. This may make the response harder to understand, but it can also introduce new terms that can be clarified and explained in future medical visits. Even so, users can provide feedback on the language and responses provided^5^, allowing AI to learn from errors and develop its own algorithms^6^. Because ChatGPT has limited access to information sources and is not connected to the internet, much of its training and refinement comes from the community that uses it, both in the quantity and quality of the data^5^ ^,^ ^12^.

AI systems are already part of everyday life and serve as an important source of innovation in health care, helping to develop new medications, support clinical decision-making, and deliver high-quality care^13^. Even so, the health care industry represents only a small fraction of the vast potential and innovative possibilities that ChatGPT promises^14^. ChatGPT has shown a promising future as a complementary tool for clarifying postoperative questions^11^. 

### Study Limitations

The main limitations of this study include the sample size, the lack of longitudinal follow-up of patients, and subjectivity in assessing the clarity of responses. In addition, the version of ChatGPT used may have limitations in the accuracy of medical responses. 

## CONCLUSION

The results of this study indicate that ChatGPT can be a useful tool in supporting patients in the postoperative period following thoracic surgery, providing high levels of satisfaction and understanding of the responses delivered. Artificial intelligence demonstrated potential to answer questions quickly and accessibly, which may complement traditional care and reduce the burden on the health care system.

However, the use of AI in patient care should be integrated into a hybrid care model in which technology supports medical expertise rather than replacing it. The data suggest that many patients still feel the need for professional validation to ensure the reliability of the information provided. In addition, the clarity of ChatGPT’s language may be affected by the amount of information presented, which calls for a more tailored approach to avoid overly extensive responses that could hinder comprehension.

As technology evolves, AI platforms may be customized to provide even more accurate responses by accounting for the patient’s clinical history. This individualization may optimize physician-patient communication, making postoperative follow-up more efficient, patient-centered, and accessible to a larger number of people. Therefore, incorporating artificial intelligence into health care represents an opportunity to enhance the patient experience and contribute to a more agile and integrated care system.

Future perspectives include the development of multicenter studies and randomized clinical trials to evaluate the impact of generative AI across different surgical specialties and clinical scenarios. The integration of these tools into electronic medical records may allow automatic cross-referencing of clinical information and the generation of responses contextualized to the patient’s profile. In addition, refining language models with personalized adaptation to health literacy levels represents an essential step to ensure effective, safe, and humanized communication between patients and technology.
